# Prevalence of Impaired Fasting Glucose and Type 2 Diabetes in Kazakhstan: Findings From Large Study

**DOI:** 10.3389/fpubh.2022.810153

**Published:** 2022-02-24

**Authors:** Binur Orazumbekova, Alpamys Issanov, Kuralay Atageldiyeva, Salim Berkinbayev, Gulnara Junusbekova, Laura Danyarova, Zhanmedet Shyman, Akmaral Tashmanova, Antonio Sarria-Santamera

**Affiliations:** ^1^Department of Medicine, School of Medicine, Nazarbayev University, Nur-Sultan, Kazakhstan; ^2^Department of Cardiology, Kazakh National Medical University, Almaty, Kazakhstan; ^3^Department of Postgraduate Education, Research Institute of Cardiology and Internal Diseases, Almaty, Kazakhstan; ^4^Department of Research and Innovations, Kazakh Medical University of Continuing Education, Almaty, Kazakhstan

**Keywords:** diabetes, impaired fasting glucose, epidemiology, risk factors, Kazakhstan

## Abstract

Type 2 diabetes mellitus (T2DM) is a serious public health problem. A large proportion of patients with T2DM are unaware of their condition. People with undiagnosed T2DM are at a greater risk of developing complications, whereas prediabetes has an elevated risk of becoming T2DM. The aim of this study is to estimate the prevalence of impaired fasting glucose (IFG), undiagnosed and prior-diagnosed T2DM in Kazakhstan. A cross-sectional study was conducted in four geographically remote regions using the WHO STEP survey instrument. The status of T2DM of 4,753 participants was determined using the WHO diagnostic criteria based on fasting plasma glucose (FPG) level. As a result, the survey-weighted prevalence of IFG was 1.9% (95% CI 1.1%; 3.5%) and of T2DM was 8.0% (95% CI 3.8; 15.9). A total of 54% of T2DM have been newly diagnosed with T2DM. Being 55–64 years old (OR = 2.71, 95% CI 1.12; 6.60) and having lowered HDL-C level (OR = 3.72, 95% CI 1.68; 8.23) were found to be independent predictors for IFG. Being older than 45 years, a female (OR = 0.57, 95% CI 0.39; 0.83), having high waist circumference, was associated with newly diagnosed T2DM. Whereas, the age older than 45 years, high waist circumference, and family history of diabetes (OR = 2.42, 95% CI 1.64; 3.54) were associated with preexisting T2DM. This study shows a high prevalence of IFG and a high proportion of newly diagnosed T2DM in Kazakhstan. A series of risk factors identified in the study may be used to strengthen appropriate identification of IFG or undiagnosed patients in healthcare settings to deliver either preventive or therapeutic interventions aimed to reduce the incidence of T2DM or the delay of their complications. Further longitudinal studies are needed to confirm these associations in our population.

## Introduction

Type 2 diabetes mellitus (T2DM) is a serious chronic disease, which affects the health and wellbeing of people and puts a tremendous burden on healthcare systems and societies worldwide (1). Half of the expenditure on diabetes care is spent to treat diabetes-related complications (2). Globally, the mortality attributable to T2DM ranges between 6.8% and 16.2% (3). It is estimated that by 2045, the number of people with T2DM could be as high as 700 million (10.9% globally). It has been estimated that half of adults living with T2DM are unaware of their condition (4). A total of 84% of them are from low- or middle-income countries (LMICs) (1). People with undiagnosed T2DM are at a greater risk of developing complications than patients with previously diagnosed and on treatment (5). Undiagnosed and not treated people are exposed to high glucose levels for a long duration, which could lead to more severe complications (6–8). People with prediabetes are at a higher risk of becoming T2DM (9). The proportion of prediabetes, which includes impaired fasting glucose (IFG) and impaired glucose tolerance, could reach an epidemic rate of 8.6% worldwide in 2045 where 72% of whom will be from LMICs (1).

Kazakhstan, a former Soviet Union country located in Central Asia, has seen an increase in the prevalence of T2DM since attaining independence. Few studies were conducted in Kazakhstan investigating the proportion of T2DM and prediabetes (10–12). The estimates from these studies show differences with the National Diabetes Registrar of Kazakhstan statistics (8.2–12.5% vs. 2.3%, respectively). Despite an introduction of the National Diabetes Screening Program, since 2011, Kazakhstan shows a relevant proportion of the population with risk factors for T2DM. It was estimated that 54% of the population in Kazakhstan is overweight or obese and 45% have dyslipidemia (cholesterol level >5.0 mmol/L) (13).

Given inconsistent data on the prevalence of T2DM and lack of understanding of distribution of risk factors for T2DM in Kazakhstan population, we aimed to determine the prevalence of IFG, undiagnosed T2DM, and prior-diagnosed T2DM and to determine the independent risk factors associated with these conditions.

## Materials and Methods

### Study Design

This cross-sectional study was based on the data from a large nationwide study, supported by the Ministry of Health, which was initiated to monitor non-communicable diseases (NCDs) in Kazakhstan (14). This study used the standardized WHO STEPwise approach to NCDs (15). This tool applies a sequential process of data collection: collecting information about risk factors using a questionnaire, physical examination, and collection of blood samples for biochemical analysis (15). To select a nationally representative sample from the population for the STEPS survey, by the recommendation of WHO, a double-stage cluster random sampling method was utilized. Four large oblasts (provinces) from the north, south, and west regions of Kazakhstan were chosen – Pavlodar, Almaty, South Kazakhstan, and Aktobe, respectively (16). At the first stage, every oblast was stratified to major cities, towns, and villages according to the size of the population. Using simple random sampling, within each stratum, one or two clusters were selected (one major city, two towns, and two villages). In the second stage, within each cluster, 100 households were randomly selected from a list of households in the area (Supplementary Figure 1). Trained interviewers collected the data by surveying face-to-face, door-to-door participants from the randomly selected households during the period of January 2015 and December 2017.

### Study Population

Our study included adults aged 18–69, who gave informed consent to participate and those who have completed data on fasting plasma glucose (FPG) level. People with type 1 diabetes or in a state of drug or alcohol intoxication, as well as those having other conditions that do not allow them to give informed consent, were excluded from the study. Overall, 4,753 participants met the inclusion criteria.

### Sample Size

The needed sample size was calculated at 95% confidence interval (CI), margin of error of 0.02, nationally estimated prevalence of T2DM of 0.024 (12), design effect of 1.5, and the number of age–sex estimates of 8 (four age groups of men and women). We used the following formula to calculate the sample size:


(1) $$\begin{array}{l} n=\frac{Z^{2}*p*\left(1-p\right)}{d^{2}}*DE*a \end{array}$$

where,

n = needed sample size.

Z = standard score (Z-score).

p = estimated prevalence of T2DM.

d = margin of error.

DE = design effect.

a = number of age-sex estimates.


$$\begin{array}{l} n=\left[(1.96^{2}*0.024*0.976\right)/0.02^{2}]*1.5*8=2,700 \end{array}$$

Thus, the needed sample size was 2,700 respondents.

### Outcome Variables

The World Health Organization (WHO) guidelines were used to define persons with IFG, newly diagnosed T2DM, and preexistingT2DM cases (17). Participants whose FPG measurements were between 6.1 and 6.9 mmol/L were defined as having IFG. Those whose FPG was higher than 7.0 mmol/L without previous history of clinically confirmed diabetes were categorized as newly diagnosed T2DM. Participants who had the previous history of diagnosed diabetes or a history of taking antidiabetic drugs with hemoglobin A1c (HbA1c) level ≥6.5 mmol/dL were considered as participants with preexisting T2DM.

### Independent Variables

Data on participants' sociodemographic characteristics, such as age (<45, 45–54, 55–64, and >64 years), sex, ethnicity (Kazakh, Russian, and others), place of residence (rural/urban), and the level of education (primary, vocational/secondary, and university), were collected by trained personnel using the WHO STEPS survey instrument (18). Participants' ethnicity was determined based on verbal self-defining or relating to a specific ethnic group during an interview. Participants were also surveyed about the history of family diabetes (Yes/No), previous history of high blood pressure (Yes/No), and use of antihypertensive drugs (Yes/No). Anthropometric characteristics, such as body mass index (BMI), waist circumference (WC), and arterial blood pressure (BP) were measured by trained interviewers. According to WHO, BMI was estimated by taking participant's weight, in kilograms, divided by participant's height, in meters squared, and ranges referred to non-obesity (<25 kg/m^2^), overweight (25–29.9 kg/m^2^), and obesity (>30 kg/m^2^), respectively (19). The WC was categorized based on WHO criteria for normal (<94 cm for men and 80 cm for women), increased (94–101.9 cm for men and 80–87.9 cm for women), and high (102 cm and higher for men and 88 cm and higher for women) measurements (20). Arterial BP measurements were recorded three times from both arms using a manual sphygmomanometer in a sitting position. The mean value of three measurements was used to determine the presence of high BP. High BP was defined as systolic BP≥140 mm Hg or diastolic BP ≥ 90 mm Hg. Upon the completion of survey and anthropometric measurements, participants were invited to the local outpatient clinic for blood sample collection at a fasting state. A qualified phlebotomist collected a blood sample. Venous blood specimen was collected in Vacutainer tubes containing ethylenediaminetetraacetic acid (EDTA). Blood samples were separated within 6 to 8 h of specimen collection and stored at 2°C−8°C temperature. The instruments were calibrated daily based on standardized procedures. Fasting blood glucose, HbA1C, total cholesterol (lower or higher than 5.2 mmol/dL), triglycerides (lower or higher than 1.7 mmol/dL), and HDL-cholesterol (HDL-C lower or higher than 1 mmol/dL) levels were measured using an automatic biochemical analyzer – Cobas Integra 400 Plus analyzer (Roche Diagnostics, Switzerland).

### Statistical Analysis

Statistical analysis was performed using STATA 15 software (21). To adjust for the complex survey design, we used survey weights, clusters, and strata in all statistical analyses (22). Continuous variables were presented using means, whereas percentages and corresponding confidence intervals were estimated for categorical variables. The unadjusted bivariable analysis was performed to find the association between groups of T2DM and other categorical variables using the Rao-Scott chi-square test, whereas the design-adjusted F-test was used to test relationships with continuous independent variables. Survey-weighted multiple logistic regression models were built for IFG vs. non-diabetes, for newly diagnosed diabetes vs. non-diabetes, and for preexisting diabetes vs. non-diabetes. Based on epidemiological reasoning, age, sex, ethnicity, and education were forced to the models, and the rest of the covariates were selected by backward elimination using the AIC-based approach. The multivariable analyses were performed on the analytic dataset with only complete responses. The discrimination of the models was assessed by calculating the area under the curve (AUC) from the receiver operating characteristic (ROC) curves. AUC close to 0.5 or lower indicated low predictive power of the model, whereas AUC > 0.7 showed moderate or high predictive power of the adjusted model.

## Results

### Descriptive Statistics

The number of participants who met the inclusion criteria was 4,753. The participants' average age was 46.5 years (95% CI 44.3 years; 48.7 years) (Table 1). The majority of participants were women (76.3%, 95% CI 72.0%; 80.1%), ethnic Kazakhs (59.6%, 95% CI 51.5%; 67.3%), overweight or obese (31.7% (95% CI 29.3%; 34.3%), and 29.5% (95% CI 25.1%; 34.2%) and lived in urban areas (52.3%, 95% CI 46.2%; 59.3%).

**Table 1:** Demographic characteristics of the study participants from Pavlodar, Almaty, South Kazakhstan, and Aktobe oblasts (2015–2017).

**Characteristics**	Nondiabetes % (95% CI)^*^, (*N* = 4,267)	IFG % (95% CI)^*^, (*N* = 114)	Newly diagnosed diabetes % (95% CI)^*^, (N = 152)	Preexisting diabetes % (95% CI)^*^, (N = 220)	***P*-value**	Total % (95% CI)^*^, (*N* = 4,753)
Age in years, mean (95% CI)	45.6 (43.8; 47.8)	51.6 (48.2; 55.1)	53.6 (50.1; 57.1)	56.1 (52.1; 60.0)	<0.001	46.5 (44.3; 48.7)
**Age**
<45 years	95.4 (90.9; 97.7)	1.3 (0.6; 2.9)	2.1 (0.9; 4.9)	1.2 (0.4; 3.2)	<0.001	41.6 (35.6; 47.8)
45–54 years	91.3 (83.4; 95.6)	1.5 (0.6; 3.7)	3.4 (1.4; 8.1)	3.8 (1.4; 9.6)		25.0 (23.5; 26.7)
55–64 years	84.1 (70.9; 91.9)	3.2 (1.8; 5.8)	7.4 (3.3; 15.9)	5.3 (1.9; 13.1)		21.0 (18.6; 23.5)
>64 years	79.9 (66.1; 89.0)	2.6 (1.2; 5.4)	8.0 (3.9; 15.9)	9.5 (4.1; 20.3)		12.4 (8.7; 17.3) (11 missing)
**Sex**
Males	89.1 (80.7; 94.1)	1.2 (0.5; 3.1)	6.4 (3.3; 12.1)	3.3 (1.1; 9.6)	0.01	23.7 (19.8; 28.0)
Females	90.4 (81.3; 95.4)	2.1 (1.1; 4.2)	3.6 (1.6; 7.5)	3.9 (1.5; 9.8)		76.3 (72.0; 80.1) (23 missing)
**Ethnicity**
Kazakh	91.1 (81.5; 96.0)	1.8 (0.9; 3.6)	3.5 (1.4; 8.6)	3.6 (1.3; 9.7)	0.47	59.6 (51.5; 67.3)
Russian	87.4 (77.8; 93.2)	2.4 (1.0; 5.4)	5.3 (2.4; 11.3)	4.9 (2.3; 10.4)		20.1 (13.7; 28.5)
Others	90.1 (83.2; 94.3)	1.8 (0.9; 3.6)	5.1 (2.6; 9.5)	3.0 (0.7; 11.7)		(45 missing)
**Residence**
Urban	90.0 (81.1; 95.0)	1.9 (1.0; 3.6)	4.4 (2.1; 8.7)	3.7 (1.4; 9.7)	0.63	52.3 (46.2; 59.3)
Rural	92.8 (89.7; 95.1)	1.8 (0.9; 3.7)	2.5 (1.2; 4.8)	2.9 (1.3; 6.3)		47.7 (41.3; 54.5) (0 missing)
**Level of education**
Primary	87.5 (79.4; 92.7)	2.3 (1.1; 4.9)	7.3 (3.9; 13.2)	2.9 (0.9; 8.4)	<0.05	22.3 (17.5; 27.9)
Secondary	89.9 (80.5; 95.1)	2.3 (1.3; 4.3)	3.9 (1.9; 8.1)	3.9 (1.3; 10.4)		40.9 (35.6; 46.4)
University	91.7 (81.5; 96.5)	1.3 (0.5; 3.3)	2.9 (1.2; 6.5)	4.1 (1.4; 11.7)		36.8 (33.5; 40.4) (52 missing)
**BMI**
<25 kg/m2	93.7 (86.8; 97.2)	1.6 (0.7; 3.7)	3.5 (1.5; 7.6)	1.2 (0.3; 4.1)	<0.001	38.8 (35.4; 42.3)
25–29.99kg/m2	90.8 (80.9; 95.8)	1.5 (0.7; 3.1)	4.3 (1.9; 9.3)	3.4 (1.2; 9.5)		31.7 (29.3; 34.3)
≥30 kg/m2	84.1 (72.3; 91.5)	2.7 (1.5; 4.8)	5.8 (2.8; 11.8)	7.4 (2.7; 18.7)		29.5 (25.1; 34.2) (159 missing)
**Waist circumference (cm)**
M: > 94; F: >80	95.0 (89.8; 97.6)	1.4 (0.7; 2.8)	2.5 (1.2; 5.2)	1.1 (0.4; 3.2)	<0.001	32.3 (29.4; 35.4)
M: 94–102; F: 80–88	90.5 (80.1; 95.8)	1.5 (0.7; 2.9)	5.7 (2.4; 12.9)	2.3 (0.7; 6.7)		21.0 (17.9; 24.4)
M: 102 <; F: 88 <	86.5 (76.2; 92.7)	2.5 (1.4; 4.6)	4.9 (2.4; 9.7)	6.1 (2.4; 15.0)		46.7 (0.41; 0.52) (0 missing)

*IFG, impaired fasting glucose; BMI, body mass index; M, male; F, female*.

* *Estimates were survey-weighted to represent the population parameters*.

**Figure 1:**
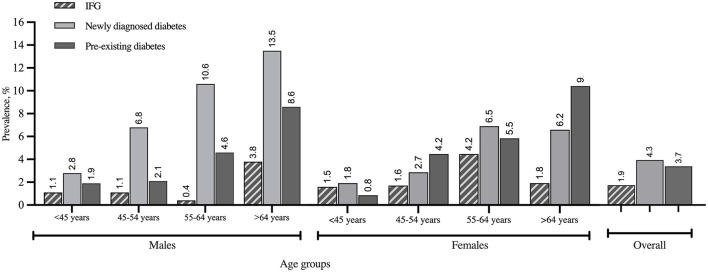
Overall and stratified by sex and age groups survey-weighted prevalence of IFG, newly diagnosed diabetes, and preexisting diabetes estimated using the cross-sectional data from Pavlodar, Almaty, South Kazakhstan, and Aktobe oblasts (2015–2017).

**Figure 2:**
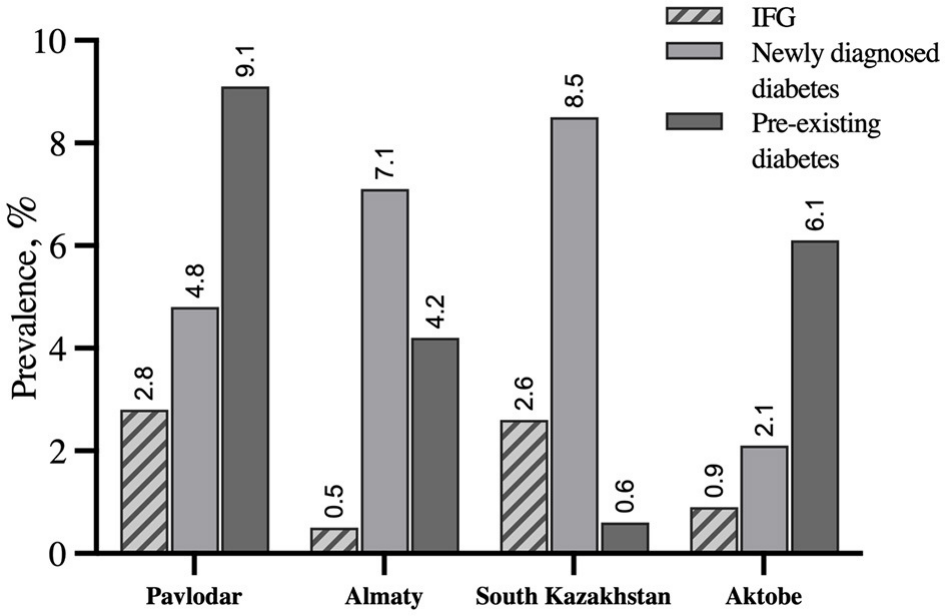
Survey-weighted prevalence of IFG, newly diagnosed diabetes, and preexisting diabetes in Pavlodar, Almaty, South Kazakhstan, and Aktobe oblasts estimated based on the cross-sectional survey data from 2015 through 2017.

The survey-weighted prevalence of IFG was 1.9% (95% CI 1.1%; 3.5%). Overall, survey-weighted prevalence of T2DM was 8.0% where 4.3% (95% CI 2.1%; 8.7%) were newly diagnosed and 3.7% (95% CI 1.4%; 9.7%) had preexisting T2DM. Stratified survey-weighted prevalence of IFG and T2DM by sex and age groups is shown in Figure 1. The highest prevalence of preexisting T2DM was determined in Pavlodar and Aktobe oblasts whereas high proportions of newly diagnosed T2DM were identified in Almaty and South Kazakhstan oblasts (Figure 2).

### Bivariable Statistical Analysis

In unadjusted analysis, the prevalence of IFG, newly diagnosed T2DM, and preexisting T2DM differed in each age category (*p* < 0.001). The highest prevalence of IFG was in the age category between 55 and 64 years (3.2%, 95% CI 1.8%; 5.8%), whereas the highest prevalence of newly diagnosed and preexisting T2DM was in the oldest age group, 8.0% (95% CI 3.9%; 15.9%) and 9.5% (95% CI 4.1%; 20.3%), respectively. The proportion of IFG and preexisting diabetes was higher among women [2.1% (95% CI 1.1%; 4.2%) and 3.9% (95% CI 1.5%; 9.8%)], whereas newly diagnosed T2DM was more prevalent among men (6.4%, 95% CI 3.3%; 12.1%).

People with IFG (2.3%, 95% CI 1.1%; 4.9%) and newly diagnosed (7.3%, 95% CI 3.9%; 13.2%) cases were frequently found among participants with primary education, whereas preexisting T2DM cases were more prevalent among participants with a university degree (4.1%, 95% CI 1.4%; 11.7%).

Impaired fasting glucose, newly diagnosed T2DM, and preexisting T2DM were higher among obese participants with BMI >30 kg/m^2^ (2.7% (95% CI 1.5%; 4.8%), 5.8% (95% CI 2.8%; 11.8%), 7.4% (95% CI 2.7%; 18.7%), *p* < 0.001) and participants with waist circumference >102 cm for men and >88cm for women for IFG and preexisting cases (2.5% (95% CI 1.4%; 4.6%) and 6.1% (95% CI 2.4%; 15.0%), whereas for patients with newly diagnosed diabetes, it was higher for people with WC 94–102 cm for men and 80–88 cm for women (5.7%, 95% CI 2.4%; 12.9%, *p* < 0.001). The proportions of newly diagnosed and preexisting T2DM were higher among participants who had high blood pressure (*p* < 0.05), who had a history of high blood pressure (*p* < 0.001), and who had a family history of diabetes (*p* < 0.001). A high proportion of participants with newly diagnosed and preexisting T2DM had elevated levels of triglycerides (*p* < 0.001), total cholesterol (*p* < 0.001), and lowered HDL-C level (*p* < 0.05) (Table 2).

**Table 2:** Physiological and biochemical characteristics of the study participants from Pavlodar, Almaty, South Kazakhstan, and Aktobe oblasts (2015–2017).

**Characteristics**	Nondiabetes % (95% CI)^*^, (*N* = 4267)	IFG % (95% CI)^*^, (*N* = 114)	Newly diagnosed diabetes % (95% CI)^*^, (N = 152)	Preexisting diabetes % (95% CI)^*^, (N = 220)	***P*-value**	Total % (95% CI)^*^, (*N* = 4753)
**High blood pressure**						
Yes	87.2 (74.0; 94.2)	1.6 (0.5; 4.7)	6.0 (2.5; 13.8)	5.2 (1.7; 14.8)	<0.05	25.2 (21.8; 29.0)
No	92.2 (82.0; 96.8)	1.8 (0.9; 3.6)	2.9 (1.1; 7.4)	3.1 (0.9; 9.7)		74.8 (71.0; 78.2) (485 missing)
**History of high blood pressure**						
Yes	85.0 (73.7; 91.9)	2.5 (1.3; 4.9)	5.7 (2.9; 10.7)	6.8 (2.8; 15.8)	<0.001	44.7 (40.1; 49.4)
No	93.8 (87.2; 97.2)	1.5 (0.7; 2.9)	3.2 (1.4; 6.9)	1.5 (0.5; 4.9)		55.3 (50.6; 59.9) (357 missing)
**Family history of diabetes**,						
Yes	86.9 (75.2; 93.5)	1.1 (0.5; 2.5)	4.8 (2.5; 9.2)	7.2 (2.5; 18.7)	<0.001	21.6 (18.5; 25.2)
No	90.9 (82.3; 95.6)	2.2 (1.1; 4.3)	4.1 (1.9; 8.7)	2.8 (1.0; 7.3)		78.4 (74.8; 81.5) (40 missing)
**Total cholesterol**						
≤5.2 mmol	92.4 (85.7; 96.1)	1.5 (0.8; 2.7)	3.6 (1.6; 7.5)	2.5 (0.8; 7.1)	<0.001	67.6 (59.8; 74.6)
>5.2 mmol	85.1 (72.3; 92.6)	2.8 (1.3; 5.8)	5.8 (2.8; 11.6)	6.3 (2.5; 14.6)		32.4 (25.4; 40.2) (23 missing)
**HDL-C level**						
≤1 mmol	91.7 (82.4; 96.3)	1.5 (0.7; 3.0)	3.5 (1.4; 8.5)	3.3 (1.3; 8.6)	<0.05	81.4 (67.3; 90.3)
> 1 mmol	82.7 (75.1; 88.3)	4.0 (2.2; 7.2)	7.8 (4.8; 12.5)	5.5 (1.6; 17.5)		18.6 (9.7; 32.7) (26 missing)
**Triglycerides**						
≤1.7 mmol	93.4 (87.0; 96.7)	1.6 (0.8; 3.2)	2.7 (1.2; 6.2)	2.3 (0.9; 5.7)	<0.001	74.9 (72.0; 77.5)
≥ 1.7 mmol	80.1 (64.6; 89.9)	2.9 (1.3; 6.1)	9.0 (4.4; 17.8)	8.0 (2.8; 20.5)		25.1 (22.5; 27.9) (25 missing)
**Fasting plasma glucose level in mmol/L, mean (95% CI)**	4.2 (3.9; 4.5)	6.4 (6.3; 6.5)	11.8 (10.9; 12.7)	6.9 (5.1; 8.6)	<0.001	4.7 (4.1; 5.2)

*IFG, impaired fasting glucose; HDL-C, high density lipoprotein cholesterol*.

* *Estimates were survey-weighted to represent the population parameters*.

As BMI and WC were highly correlated (*r* = 0.75), to avoid the effect of multicollinearity in multivariable analysis, only WC, which is known as a better indicator of adipose tissue than BMI, was considered in the modeling (23). Additionally, the level of triglycerides was collinear with WC, which significantly lowers the effect sizes and standard errors, so the odds ratios (OR) of triglyceride levels were taken from separate models.

### Multivariable Model of IFG

The adjusted model for prediabetes (AUC = 0.72) included age, sex, ethnicity, WC, place of residence, and HDL-C level (Table 3). Participants aged 55–64 years had higher odds to have IFG than younger individuals (OR = 2.71, 95% CI 1.12; 6.60). Low level of HDL-C was associated with higher odds for having IFG (OR = 3.72, 95% CI 1.68; 8.23). The results of AUCs are shown in Supplementary Figures 2–4.

**Table 3:** Multivariable logistic regression models of risk factors for IFG, newly diagnosed diabetes, and preexisting diabetes (vs. non-diabetes) using the cross-sectional data from Pavlodar, Almaty, South Kazakhstan, and Aktobe oblasts (2015–2017).

**Risk factors**	**IFG, OR (95% CI)**	**Newly diagnosed diabetes, OR (95% CI)**	**Preexisting diabetes, OR (95% CI)**
**Age**
<45 years	Ref.	Ref.	Ref.
45–54 years	1.07 (0.43; 2.63)	1.85 (1.09; 3.14)^*^	3.10 (2.58; 3.73)^***^
55–64 years	2.71 (1.12; 6.60)^*^	3.86 (2.52; 5.92)^***^	4.67 (2.79; 7.82)^***^
>64 years	1.74 (0.57; 5.31)	3.92 (1.27; 12.06)^*^	8.94 (5.20; 15.35)^***^
**Sex**
Males	Ref.	Ref.	Ref.
Females	1.70 (0.59; 4.86)	0.57 (0.39; 0.83)^**^	0.87 (0.57; 1.34)
**Ethnicity**
Kazakh	Ref.	Ref.	Ref.
Russian	1.08 (0.45; 2.61)	1.05 (0.57; 1.93)	0.76 (0.44; 1.37)
Other	0.67 (0.38; 1.21)	0.93 (0.45; 1.94)	0.64 (0.25; 1.63)
**Waist circumference**
M: >94 cm; F: >80 cm	Ref.	Ref.	Ref.
M: 94–102 cm; F: 80–88 cm	0.76 (0.40; 1.47)	1.94 (1.53; 2.47)^***^	1.47 (0.99; 2.18)
M: 102 cm <;F: 88 cm <	1.15 (0.74; 1.77)	1.58 (1.02; 2.48)^*^	3.02 (2.16; 4.20)^***^
**History of family diabetes**
No	-	-	Ref.
Yes			2.42 (1.64; 3.54)^***^
**Place of residence**
Urban	Ref.	Ref.	Ref.
Rural	0.62 (0.22; 1.75)	0.44 (0.20; 0.95)^*^	0.54 (0.10; 2.85)
**HDL-C**
>1 mmol	Ref.	Ref.	Ref.
≤1 mmol	3.72 (1.68; 8.23)^**^	2.20 (0.91; 5.28)	1.91 (0.92; 3.96)

* *(p < 0.05)*;

** *(p < 0.001)*;

*** *(p < 0.0001)*.

*OR, odds ratio; CI, confidence interval; IFG, impaired fasting glucose; M, male; F, female; HDL-C, high-density lipoprotein cholesterol; Ref, reference group*.

*Additionally, models were adjusted for education levels*.

### Multivariable Model of Newly Diagnosed T2DM

The adjusted model for newly diagnosed T2DM (AUC = 0.74) included age, sex, ethnicity, WC, place of residence, and HDL-C levels (Table 3). Older people tended to have higher odds of newly diagnosed with T2DM compared with those who were younger ones. In comparison with men, women had 43% lower odds (OR = 0.57, 95% CI 0.39; 0.83) of having newly diagnosed with T2DM. Additionally, participants with WC 94–102 cm for men and WC 80–88 cm for women and WC >102 cm for men and >88 cm for women had higher odds to have newly diagnosed diabetes than people with normal ranges of WC. Urban residents had higher odds to be newly diagnosed with T2DM (OR = 2.28, 95% CI 1.05; 4.97) than rural residents.

### Multivariable Model of Preexisting Diabetes

The adjusted model for preexisting T2DM (AUC = 0.78) included age, sex, ethnicity, WC, family history of diabetes, place of residence, and HDL-C level (Table 3). The odds to have preexisting T2DM were increasing with age. According to WC, the odds of having preexisting T2DM were higher for obese people (OR = 3.02, 95% CI 2.16; 4.20). People with preexisting T2DM were likely to report a family history of diabetes (OR = 2.41, 95% CI 1.64; 3.54).

## Discussion

Our study based on a multistage cluster random sampling in Kazakhstan determined that the prevalence of IFG was 1.9% and T2DM was 8.0%. A total of 54% of T2DM cases were not previously diagnosed. Similar results were obtained from a previous study where authors reported the prevalence at 8.2% (95% CI 7.7%−8.6%) (10). A bit higher estimate (12.5%) was previously found in Astana city (capital, pop. size 1 million) (11). However, these studies had limitations in the sampling approach and were limited to one location study design. The International Diabetes Federation (IDF) reported a prevalence of 7.0% (95% CI 4.8–11.0) (24). However, this estimation was based on the extrapolation from neighboring countries in the region given the lack of national estimates and nonexistence of a unified electronic T2DM registry in Kazakhstan (24).

Based on our results and extrapolation, we estimate that almost 1.4 million Kazakhstan people could have T2DM and more than 340 thousand could have IFG condition. It seems that the government estimation is much lower: it is reported to be 423.4 thousand of diagnosed T2DM cases and no data on people with prediabetes, especially IFG, which indicates potentially underreporting of T2DM prevalence in Kazakhstan (16, 25). To be more robust in our estimations, we used the WHO criteria for IFG (6.1–6.9 mmol/dL) whereas in case of applying the threshold of American Diabetes Association (5.6–6.9 mmol/dL), the proportion of IFG could go as high as 5.4% (95% CI 3.5–8.1) (17, 26). Evidence suggests that people with prediabetes have an elevated risk to develop diabetes and other microvascular or macrovascular complications and therefore should be screened every 3 years (26).

According to the results of the National Screening Program in 2016, more than 1.3 million people were screened for diabetes and 0.6% new cases were identified (27). People aged 40 years and older were allowed for a free screening, and they were recruited through announcements on official websites of primary healthcare organizations (28). It is likely that at the initial stage, the National Screening Program focused on the whole population and not reached those who are at high risk for diabetes development. Lately, people who were considered at high risk based on BMI or glucose level, without applying risk scoring methods for diabetes, were referred to “Health schools” where they receive counseling on healthy lifestyle for the prevention of further disease development, but the effectiveness and adherence of such a single visit prevention program has not been assessed. Overall, the diagnosis methods and care of chronic disease as diabetes are not properly monitored.

The multivariable models of our study show good predictive capacity based on AUCs, to predict IFG, newly diagnosed T2DM, and preexisting T2DM cases. Given Kazakhstan is a diverse multiethnic country, our results did not find an association between ethnicity and T2DM. A high prevalence of T2DM among Russians was observed in a previous cross-sectional study in Astana city (11), which was explained by possible socioeconomic differences among ethnicities of urban residents (29, 30).

Older age, particularly 55–64 years for IFG and >55 years for both newly diagnosed and preexisting T2DM, was a significant predictor, with higher odds among diagnosed participants, who had the highest mean age among three groups. Previous studies have reported that older people who have more comorbidities and exposed to risk factors than younger ones tend to become aware of having diabetes when they are treated for one of those comorbidities, which may explain the difference in the effect sizes between newly diagnosed and preexisting T2DM cases (31, 32). However, men independently had higher odds to be newly diagnosed. Evidence suggests that men tend to seek medical care in later stages of disease, and are less likely to request health advice (33, 34). Family history of diabetes, which suggests genetic predisposition, was a significant predictor only for participants with preexisting T2DM, but a relationship between being aware of their condition in proclaiming a family history of the disease has been shown as well in other health conditions (35, 36).

High WC was associated with newly diagnosed and preexisting T2DM, whereas a low level of HDL-C was associated with IFG, high WC is a well-known risk factor for diabetes (23). Insulin resistance of T2DM development appears even before a person became obese, with the presence of hypertriglyceridemia (37). Thus, most of the triglycerides became “triglycerides rich in HDL” particles, exacerbating the insulin resistance state in the organism (38). This may explain the higher odds for having IFG among people with lowered HDL-C levels in the multivariable model (39–42). Previous studies identified the same trend for patients with preexisting T2DM, but the non-significance of HDL-C level for patients with preexisting diagnosed could be explained by statin use, which were not considered in our study (43, 44).

Our study determined differences in the rural-urban distribution of newly diagnosed T2DM in Kazakhstan. Residents living in urban areas had higher odds of being newly diagnosed T2DM than rural ones. Rapid urbanization in many developing countries, including Kazakhstan, was associated with the adaptation of Western lifestyle leading to increase proportions of obese populations (45, 46).

Several study limitations should be mentioned. The main limitation of our study is that the cross-sectional study design does not allow us to determine causal associations between T2DM and observed risk factors found in our study population. Second, important variables, such as dietary and physical activity characteristics, had too many missing data, and they were not included in the final analysis. Next, since data on risk factors were self-reported, there could be some inaccuracies, recall, or misclassification biases in estimating associations of the factors. Finally, no information was collected on medications that respondents were taking, whereas some medications could potentially interact with physiological or biochemical indicators.

Meanwhile, our study has some strengths, as well. The sample size was sufficiently large to detect statistically significant results. Since respondents were selected using a multi-stage cluster random sampling technique and analyses were adjusted for the complex survey design, we are convinced that the obtained results could be generalized to the whole population. Also, our study used 2 biochemical test measurements to diagnose T2DM, whereas most of the previous studies used a single biochemical measurement (10, 47–49), which has its own limitations.

## Conclusion

Our study shows a high prevalence of IFG and that a high proportion of T2DM remains undiagnosed in Kazakhstan. The analysis conducted with a nationally representative sample has identified a series of variables that may be used to strengthen early diagnostics to deliver either preventive or therapeutic interventions aimed to reduce the incidence of T2DM or the delay of their complications. Overcoming these deficiencies will require nothing less than a transformation from a system that is essentially reactive to one that is proactive. The chronic care model identifies the essential elements of a healthcare system that encourage high-quality chronic disease care. These elements are the community, the health system, self-management support, delivery system design, decision support, and clinical information systems. Evidence-based change concepts under each element, in combination, foster productive interactions between informed patients who take an active part in their care and providers.

## Data Availability Statement

The raw data supporting the conclusions of this article will be made available by the authors, without undue reservation.

## Ethics Statement

The study involving human participants were reviewed and approved by the Institutional Research Ethics Committee of Nazarbayev University (NUSOM-IREC-NOV-2019-#19) and the Board of Ethics of the National Institute of Cardiology and Internal Diseases of Kazakhstan (Protocol#18 dated 28.01.2015).

## Author Contributions

BO, KA, SB, GJ, AI, and AS-S contributed to concept and design. KA, LD, ZS, and AT contributed to data collection. BO, KA, AI, and AS-S contributed to data analysis, interpretation, and drafting the manuscript. BO, AI, and AS-S finally revised the manuscript. All authors have approved the final version of the manuscript.

## Funding

The study design and data collection were supported by the Ministry of Education and Science of the Republic of Kazakhstan (Grant #48973/PCF-MON-OT-17). The data analysis and manuscript writing were supported by the Nazarbayev University (Faculty Development Research Grant Program FDCRGP Reference: 080420FD1916, 2020-2022).

## Conflict of Interest

The authors declare that the research was conducted in the absence of any commercial or financial relationships that could be construed as a potential conflict of interest.

## Publisher's Note

All claims expressed in this article are solely those of the authors and do not necessarily represent those of their affiliated organizations, or those of the publisher, the editors and the reviewers. Any product that may be evaluated in this article, or claim that may be made by its manufacturer, is not guaranteed or endorsed by the publisher.

## Abbreviations

T2DM: type 2 diabetes mellitus
WHO: World Health Organization
FPG: fasting plasma glucose
HbA1c: glycated hemoglobin
LMICs: low- or middle-income countries
NCDs: non-communicable diseases
BMI: body mass index
HDL-C: high-density lipoprotein cholesterol
AUC: area under the curve.
