# Supplementary material for: Prevalence of Impaired Fasting Glucose and Type 2 Diabetes in Kazakhstan: Findings From Large Study
Source: Front Public Health. 2022 Feb 24;10:810153. doi: 10.3389/fpubh.2022.810153 (PMC8907545; doi:10.3389/fpubh.2022.810153)
Supplement: Supplementary file 1 [file Data_Sheet_1.docx]

Supplementary Materials

Supplementary Figure 1. Flow diagram of inclusion and exclusion of study participants from four oblasts in Kazakhstan.


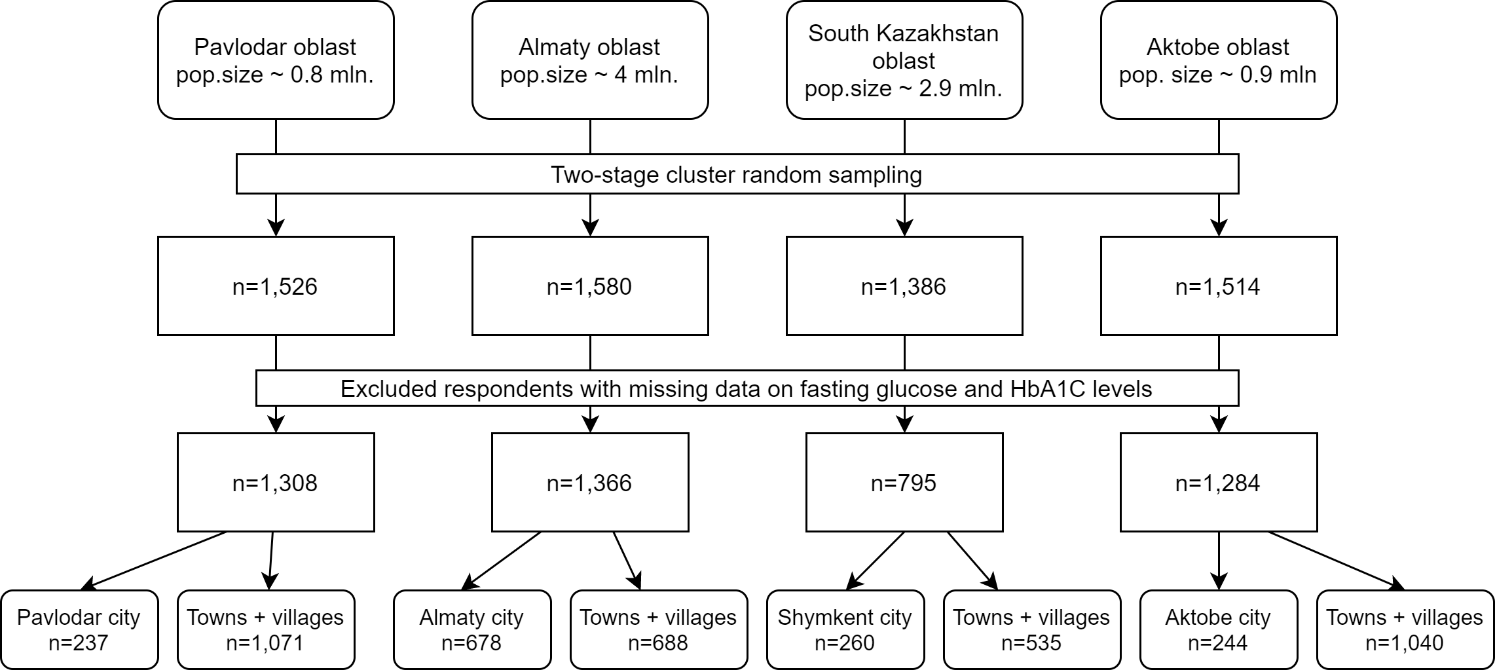


## Supplementary Figure 2. ROC/AUC for the logistic model predicting impaired fasting glucose, (AUC=0.72).


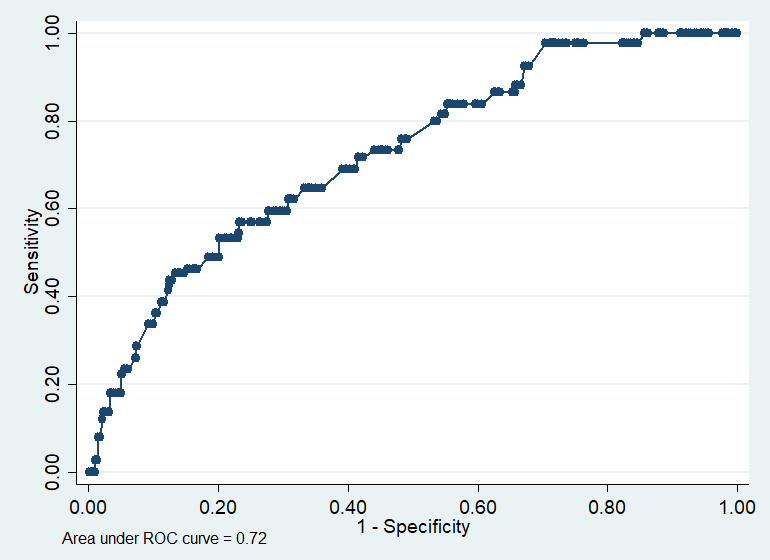


**Supplementary Figure 3. ROC/AUC for the logistic model predicting newly diagnosed DM with statistically significant variables, (AUC 0.74).**


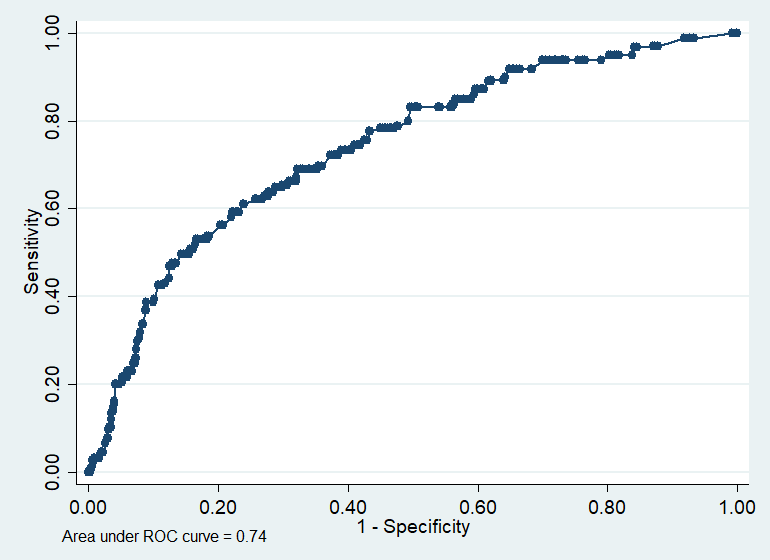


## Supplementary Figure 4. ROC/AUC for model predicting prior diagnosed DM with statistically significant variables, (AUC=0.78).


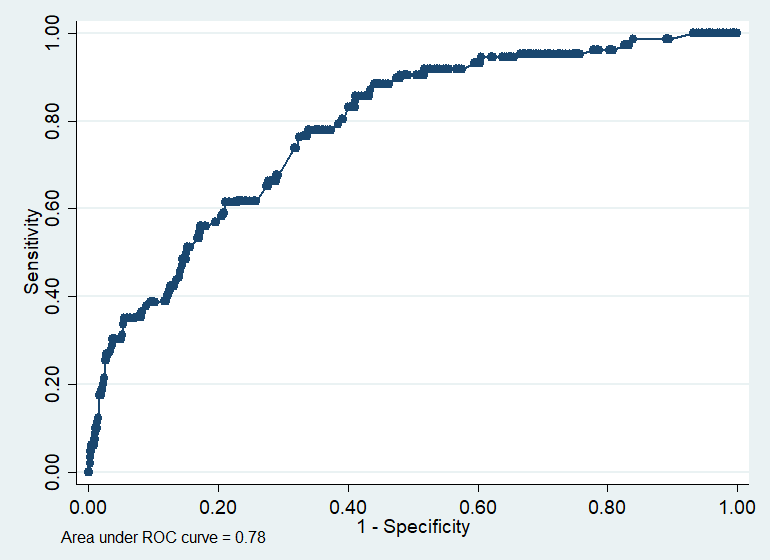


**
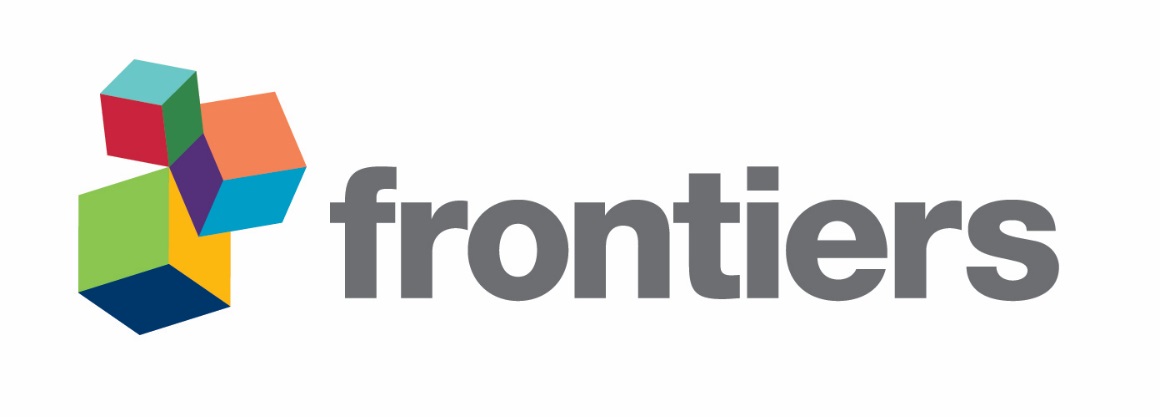
**
